# Stigmatizing and Positive Language in Birth Clinical Notes Associated With Race and Ethnicity

**DOI:** 10.1001/jamanetworkopen.2025.9599

**Published:** 2025-05-13

**Authors:** Ismael Ibrahim Hulchafo, Jihye Kim Scroggins, Sarah E. Harkins, Hans Moen, Michele Tadiello, Kenrick Cato, Anahita Davoudi, Dena Goffman, Janice James Aubey, Coretta Green, Maxim Topaz, Veronica Barcelona

**Affiliations:** 1 Columbia University School of Nursing, New York, New York; 2 University of North Carolina at Chapel Hill School of Nursing, Chapel Hill; 3 Department of Computer Science, Aalto University, Espoo, Finland; 4 Center for Community-Engaged Health Informatics and Data Science, Columbia University Irving Medical Center, New York, New York; 5 University of Pennsylvania School of Nursing, Philadelphia; 6 VNS Health, New York, New York; 7 Department of Obstetrics and Gynecology, Columbia University, New York, New York; 8 New York-Presbyterian, New York, New York

## Abstract

**Importance:**

Language used in clinical documentation can reflect biases, potentially contributing to health disparities. Understanding associations between patient race and ethnicity and documentation of stigmatizing and positive language in clinical notes is crucial for addressing health disparities and improving patient care.

**Objective:**

To examine associations of race and ethnicity with stigmatizing and positive language documentation in clinical notes from hospital birth admission.

**Design, Setting, and Participants:**

This cross-sectional study included birthing patients at 2 metropolitan hospitals in the Northeastern US between 2017 and 2019. Eligible participants were admitted for labor and birth and had at least 1 free-text clinical note. Analysis was conducted using natural language processing. Data were analyzed between March and December 2024.

**Exposures:**

Patient race and ethnicity, categorized into mutually exclusive groups of Asian or Pacific Islander, Black, Hispanic, and White.

**Main Outcome and Measures:**

Presence of 4 stigmatizing language categories (marginalized language or identities, difficult patient, unilateral or authoritarian decisions, and questioning patient credibility) and 2 positive language categories (preferred and/or autonomy, power and/or privilege).

**Results:**

Among the 18 646 patients included in the study (mean [SD] age, 30.5 [6.2] years), 2121 were Black (11.4%), 11 078 were Hispanic (59.4%), and 4270 were White (22.9%). The majority (10 559 patients [56.6%]) were insured by Medicaid. Compared with White patients, Black patients had higher odds of having any stigmatizing language (model 2: odds ratio [OR], 1.25; 95% CI, 1.05-1.49; *P* < .001), after adjustment for demographic characteristics. Black patients also had higher odds of any positive language documented (model 2: OR, 1.18; 95% CI, 1.05-1.32; *P* = .006). Hispanic patients had lower odds of documented positive language (model 2: OR, 0.90; 95% CI, 0.82-0.99; *P* = .03). Asian or Pacific Islander patients had lower odds of language documented in the power and/or privilege category (model 2: OR, 0.71; 95% CI, 0.57-0.88; *P* = .002).

**Conclusions and Relevance:**

In this cross-sectional study examining clinical notes of 18 646 patients admitted for labor and birth, there were notable disparities in how stigmatizing and positive language was documented across racial and ethnic groups. This underscores the necessity for improving documentation and communication practices to reduce the use of stigmatizing language.

## Key Points

**Question:**

Does documentation of stigmatizing and positive language vary by race and ethnicity in clinical notes from hospital birth admissions?

**Findings:**

In our cross-sectional study examining clinical notes of 18 646 patients admitted for labor and birth, Black patients had 22% higher odds of stigmatizing language documented than White patients. Hispanic patients had 10% lower odds of positive language documented than White patients.

**Meaning:**

These results document that stigmatizing and positive language differs by racial and ethnic group, suggesting that further training may be warranted to promote more equitable clinical documentation practices.

## Teaser

This cross-sectional study of adults giving birth at 2 US hospitals examines the association of stigmatizing and positive language in clinical patient notes with race and ethnicity.

## Introduction

Racial and ethnic disparities in perinatal health outcomes persist as a significant issue in the US. In labor and birth settings, racially and ethnically marginalized patients continue to experience higher rates of adverse pregnancy outcomes, such as preterm birth, severe maternal morbidity, and mortality compared with White patients.^1,2,3^ Researchers have identified racism as a leading cause of these disparities, operating at multiple societal levels through interpersonal and structural mechanisms.^4,5,6,7^ Clinicians can perpetuate racism in health care settings through explicit discrimination and subtle biases,^8^ affecting clinical judgment and leading to differential treatment.^9,10,11,12^ For example, implicit biases stemming from the false belief that Black patients have a lower pain threshold than White patients have contributed to racial and ethnic disparities in the use of neuraxial analgesia during labor.^13^

Stigmatizing and positive language documented in the electronic health record (EHR) may also convey explicit or implicit biases that can reinforce socially constructed power dynamics and disproportionately affect individuals with marginalized identities.^14^ Researchers have identified several categories of stigmatizing language in clinician documentation, including using quotations to indicate disbelief in the patient, labeling patients as difficult, expressing disapproval of patient actions, and centering clinician authority.^12,15^ Positive language may also communicate implicit biases by documenting characteristics clinicians view favorably, for example, highlighting somebody’s high income.^12,16^ Documentation of stigmatizing and sometimes positive language in the EHR may perpetuate biases and negative attitudes among clinicians, resulting in differential treatment.^17^

Importantly, studies have shown that Black patients were more likely to have stigmatizing language documented in their EHR compared with White patients in ambulatory and internal medicine health care settings.^18,19,20^ For example, Black patients were more likely to have language around noncompliance and financial difficulty than White patients in emergency settings.^21^ Additionally, another study showed that Hispanic patients were less likely to have positive language documented in their EHR compared with White patients in inpatient or outpatient settings.^18,22^ However, little is known about the association between patient race and ethnicity and documentation of stigmatizing and positive language in labor and birth settings. This is a critical gap, considering perinatal health disparities are rooted in racism and discrimination. Examining the language used in clinical notes from hospital birth admissions across patients of different racial and ethnic groups would improve our understanding of how language may reflect and perpetuate biases and disparities in labor and birth settings.

Another important knowledge gap is that existing natural language processing (NLP) studies identifying both stigmatizing and positive language in clinical documentation have relied on keyword word-search analysis.^18,19,20,23^ These approaches focus on analyzing individual words and short phrases or rely on a predefined list of words,^24^ which may not optimally capture the complex nuances of stigmatizing language. For example, the phrase “emotionally distraught” could be interpreted as judgmental in one context (eg, “Patient emotionally distraught and refused any intervention”) but empathetic in another (eg, “Patient was emotionally distraught due to recent loss and was offered additional support”). We have developed several NLP text classification models, including context-level models based on deep learning, enabling comprehensive examination of nuanced stigmatizing and positive language.^25^ This study examined associations between patient race and ethnicity and the documentation of stigmatizing and positive language in clinical notes from hospital birth admissions, applying our best-performing, newly developed NLP model—a fine-tuned version of ClinicalBERT.

## Methods

### Data and Study Settings

We studied EHR notes from patients at 2 major metropolitan hospitals in the Northeastern US between 2017 and 2019, and conducted this cross-sectional study in 2024. We included all patients greater than 20 weeks’ gestation who were admitted for birth. We analyzed 22 distinct clinical note types containing free-text narratives (eTable 1 in Supplement 1). We excluded medication orders, administration records, template-based fall assessment documents, and other structured note types from analyses. The final dataset comprised 206 248 clinical notes from 18 646 patients. To prepare the selected clinical notes for analysis, a series of preprocessing techniques were applied, including using regular expressions to extract free-text segments, removal of extraneous symbols, appropriate handling of abbreviations, and normalizing capitalization across all documents. As this was a secondary analysis of EHR data, no informed consent was obtained. This research was conducted in compliance with ethical guidelines and received approval from the institutional review board at Columbia University Medical Center.

### Primary Exposure and Outcome Variables

Patient race and ethnicity were obtained from EHR and were recorded by administrative staff; however, 45% of race and ethnicity data were missing. We imputed the missing race and ethnicity using the Bayesian Improved First Name Surname Geocoding (BIFSG) algorithm.^26^ The BIFSG algorithm imputes race and ethnicity based on first name, surname, and census block data with high accuracy.^27^ This BIFSG algorithm categorizes race and ethnicity into 6 mutually exclusive groups: American Indian or Alaskan Native, Asian or Pacific Islander, Black, Hispanic, multiracial, and White. As Hispanic is an ethnicity, patients may have identified with any race, and those in the other racial categories were not Hispanic. American Indian or Alaskan Native and multiracial categories were excluded from the analysis due to the small sample size. In our previous study, the BIFSG algorithm performed well to accurately classify race and ethnicity with C-statistics ranging from 0.93 to 0.97 for the 4 race and ethnicity groups included in the current study.^26^ The BIFSG algorithm also resulted in less biased estimates of preterm birth than complete case analysis or multiple imputation.^26^

Our study outcomes included 6 main language categories, divided into stigmatizing and positive language. Stigmatizing language is defined as language that can convey negative meanings, which may reflect writer’s implicit or explicit bias and can also transmit bias about the patient to the next readers.^14,28^ Each language category, its definition, and examples from EHR notes are presented in Table 1. These categories were determined based on previous research, including qualitative studies and NLP development.^15,29^ The stigmatizing language category comprised 4 subcategories: marginalized language or identities, difficult patient, unilateral or authoritarian decisions, and questioning patient credibility. The positive language category included preferred language and/or respecting patient autonomy (preferred and/or autonomy) and power or privilege language.

**Table 1.** Description of Stigmatizing and Positive Language Categories

Categories	Definition	Example	Model performance
**Stigmatizing language**			
Marginalized language/identities	A compilation of societal and behavioral elements that may lead to the marginalization of a particular demographic, as perceived by health care professionals to be unfavorable or problematic	“Her pregnancy is notable for scant prenatal care and use of cocaine and THC [tetrahydrocannabinol] during this pregnancy”; “Antepartum course notable for marijuana use this pregnancy”; “Patient is a 32 [year old] Dominican unmarried unemployed female”	F1 = 0.9; precision = 0.9; recall = 0.9
Difficult patient	Instances where patients disregard, reject, or fail to follow prescribed medical instructions, treatments, or services, exhibiting behaviors that do not align with health care professionals’ recommended course of action	“Pt [patient] emotionally distraught and refused any intervention”; “Complained of itching on palms/soles”; “Poor effort with pushing”	F1 = 0.8; precision = 0.8; recall = 0.8
Unilateral or authoritarian decisions	Reinforces the medical professional’s dominance in health care decision-making, emphasizing a top-down approach that prioritizes the practitioner’s perspective over patient autonomy	“Received patient awake and alert ambulatory in room”; “Pt [patient] instructed to never sleep in bed with infant for risk of suffocation and or falls”; “SW [social worker] has advised [patient] that if there continues to be yelling in room that ACS [adult and child services] will need to be contacted. No report as of yet”	F1 = 0.7; precision = 0.7; recall = 0.7
Questioning patient credibility	Skepticism toward or dismissal of a patient’s self-reported medical history or current health condition	“States she tested neg ppd this pregnancy-this info did not come from her faxed records”; “Reports normal FTS [first trimester screening] but this was not able to be confirmed by her provider”; “Unsure if patient telling the truth”	F1 = 0.6; precision = 0.6; recall = 0.6
**Positive language**			
Preferred language and/or respecting patient autonomy	Portrays individuals giving birth as active decision-makers in their childbirth experience; utilizes language that impartially captures the patient’s viewpoint	“Patient prefers depo [Depo-Provera] at pp [post-partum] appt [appointment]”; “On exam patient endorses numbness across entirety of bilateral buttocks”; “Pt [patient] declines epidural at this time-trying to deliver without analgesia”	F1 = 0.9; precision = 0.9; recall = 0.9
Power and/or privilege	Power and privilege markers indicating psychological or socioeconomic position	“FOB [father of the baby] works for [a prominent company]”; “Patient reports having a strong social/emotional marriage”; “Pt [patient] states urology resident here at [hospital]”	F1 = 0.8; precision = 0.8; recall = 0.8

### NLP Approach

We applied our previously developed NLP text classification, which demonstrated good accuracy (overall F1 score = 0.8; precision = 0.8; recall = 0.8) in a prior study, to sentences extracted from the clinical notes of 18 646 patients.^25^ NLP performance of each language category can be seen in Table 1. We did not exclude descriptors regarding patient’s family, particularly around a patient’s spouse or partner, as they can reflect bias. For example, “Pt [Patient] reports having a nurturing 2yr [year] relationship with FOB [father of the baby]...who is employed in construction,” and, “She is still married to her husband...who was reportedly abusive physically and verbally.” Following our sentence-level categorization, we aggregated the data to identify different language categories at the note and patient levels. We determined which language categories were present for each clinical note, providing a document-specific view of language use. Similarly, at the patient level, we identified language categories across all notes associated with each patient. This approach allowed us to create a comprehensive profile of the language used for each patient’s medical record, capturing the overall patterns of language categories in their documentation. We then conducted association analyses at the patient level, exploring potential relationships between the observed language patterns and race and ethnicity.

### Statistical Analysis

We conducted multivariable logistic regression analyses to examine the relationships between patient race and ethnicity and language categories. We built 4 models that included different sets of covariates with conceptual similarity. Model 1 only included race and ethnicity to show unadjusted associations. In model 2, we added demographic characteristics (maternal age, marital status, insurance type, preferred spoken language). Model 3 comprised variables from model 2 as well as birth characteristics (eg, parity, mode of birth, gestational age). Finally, model 4 included model 3 covariates as well as clinical risk factors or comorbidities (eg, preeclampsia, gestational diabetes, body mass index). Covariates were selected based on bivariate analyses examining associations between potential covariates and both exposure and outcome variables (eTable 2 in Supplement 1). We also assessed multicollinearity among the potential covariates using a correlation matrix and variance inflation factor (eTable 3, eTable 4 in Supplement 1). In addition, we conducted subgroup analyses divided by the median length of inpatient stay for birth (less than 3 days vs 3 or more days), as this may have affected amounts of stigmatizing and positive language documented. The goodness-of-fit of the logistic regression models was assessed using the Hosmer-Lemeshow test and Mean Pearson Residuals.^30,31^ Odds ratios (ORs) with 95% CIs were calculated to quantify these associations. We used an α of .05 to indicate statistical significance, and all analyses were performed using Python in Jupyter Lab version 3.0 (Jupyter Development Team).

## Results

A total of 18 646 patients were in our analytic sample (mean [SD] age, 30.5 [6.2] years) (Table 2). Most patients were White (4270 [22.9%]), 2121 patients (11.4%) were Black, and the majority of patients (11 078 [59.4%]) were Hispanic (of any race). Over two-thirds of patients were between 20 and 34 years old (12 822 [68.8%]), and approximately one quarter were 35 years old or older (5153 [27.6%]). Most patients were insured by Medicaid (10 559 [56.6%]), and the majority of patients were single (10 640 [57.1%]).

**Table 2.** Description of Study Patients’ Characteristics

Characteristics	Patients, No. (%) (N = 18 646)
Race and ethnicity	
Asian or Pacific Islander	1177 (6.3)
Black	2121 (11.4)
Hispanic	11 078 (59.4)
White	4270 (22.9)
Maternal age	
13-19 y	671 (3.6)
20-34 y	12 822 (68.8)
≥35 y	5153 (27.6)
Marital status	
Single	10 640 (57.1)
Married	7553 (40.5)
Divorced	89 (0.5)
Widowed	11 (0.1)
Other^a^	353 (1.9)
Insurance type	
Medicaid	10 559 (56.6)
Private	8087 (43.4)
Preferred spoken language	
English	11 027 (59.1)
Another language	7619 (40.9)
Mode of birth	
Vaginal	13 768 (73.8)
Cesarean	4878 (26.2)
Gestational age at birth	
Term (≥37 wks)	16 600 (89.0)
Preterm (<37 wks)	2034 (10.9)

^a^ Unspecified or unknown status.

Overall, nearly half of patients (9194 [49.3%]) had at least 1 category of stigmatizing language documented in their clinical notes (Table 3). The most frequently documented stigmatizing language category across all patients was difficult patient (5328 [28.6%]). We also found that positive language was documented in over half of all patient notes (10 948 [58.7%]). Of note, the questioning patient credibility category was rarely observed in less than 1% of all patients (16 [0.1%]). Black patients had the highest frequency of having any category of stigmatizing language documented in their notes (1164 of 2121 [54.9%]), and difficult patient language (700 of 2121 [33.0%]). Language representing the marginalized language or identities category (253 of 2121 [11.9%]) and the preferred and/or autonomy category was frequently documented among Black patients (1302 of 2121 [61.4%]).

**Table 3.** Frequencies of Stigmatizing and Positive Language by Patient Race and Ethnicity

Language categories	Patients, No. (%)^a^				
	Total (N = 18 646)	Asian or Pacific Islander (n = 1177)	Black (n = 2121)	Hispanic (n = 11 078)	White (n = 4270)
Any stigmatizing language^b^	9194 (49.3)	534 (45.4)	1164 (54.9)	5503 (49.7)	1993 (46.7)
Marginalized language or identities	1692 (9.1)	46 (3.9)	253 (11.9)	1172 (10.6)	221 (5.2)
Difficult patient	5328 (28.6)	334 (28.4)	700 (33.0)	3076 (27.8)	1218 (28.5)
Unilateral or authoritarian decisions	4860 (26.0)	304 (25.8)	616 (29.0)	2791 (25.2)	1139 (26.7)
Questioning patient credibility	16 (0.1)	0	2 (0.1)	10 (0.1)	4 (0.1)
Any positive language^b^	10 948 (58.7)	686 (58.3)	1378 (64.9)	6381 (57.6)	2503 (58.6)
Preferred language and/or respecting patient autonomy	8215 (44.1)	649 (55.1)	1302 (61.4)	6171 (55.7)	2309 (54.1)
Power and/or privilege	1741 (9.3)	111 (9.4)	284 (13.4)	785 (7.1)	561 (13.1)

^a^ The sum of the percentages is greater than 100 because language categories are not mutually exclusive.

^b^ Any instances of language subcategories.

In the full sample of 18 646 patients, Black patients were significantly more likely to have any stigmatizing or any positive language than those in other racial and ethnic groups in all models: the unadjusted model 1 (stigmatizing language: OR, 1.39; 95% CI, 1.25-1.54; positive language: OR, 1.31; 95% CI, 1.18-1.46), model 2 controlling for demographics (stigmatizing language: OR, 1.22; 95% CI, 1.25-1.54; positive language: OR, 1.18; 95% CI, 1.05-1.32), and model 3 controlling for demographics and birth characteristics (stigmatizing language: OR, 1.16; 95% CI, 1.03-1.31; positive language: OR, 1.20; 95% CI, 1.06-1.18) (Table 4). Black patients were also more likely than White patients to have any stigmatizing language documented adjusting for demographics in both length-of-stay subgroups: 3 days or less (OR, 1.25; 95% CI, 1.05-1.49; *P* = .01) and 3 days or more (OR, 1.21; 95% CI, 1.04-1.40; *P* = .01).

**Table 4.** Unadjusted and Adjusted Logistic Regression Models Examining Any Stigmatizing or Positive Language by Patient Race and Ethnicity

Models, by race and ethnicity	Any stigmatizing language, OR (95% CI)^a^	Any positive language, OR (95% CI)^a^
**Full sample (N = 18 646)**		
Asian or Pacific Islander		
Model 1^b^	0.95 (0.83-1.08)	0.99 (0.86-1.12)
Model 2^c^	0.95 (0.83-1.08)	1.00 (0.88-1.14)
Model 3^d^	0.92 (0.80-1.05)	1.03 (0.90-1.18)
Black		
Model 1^b^	1.39 (1.25-1.54)	1.31 (1.18-1.46)
Model 2^c^	1.22 (1.09-1.37)	1.18 (1.05-1.32)
Model 3^d^	1.16 (1.03-1.31)	1.20 (1.06-1.35)
Hispanic		
Model 1^b^	1.13 (1.05-1.21)	0.96 (0.89-1.03)
Model 2^c^	0.97 (0.89-1.07)	0.90 (0.82-0.99)
Model 3^d^	0.96 (0.87-1.06)	0.94 (0.85-1.04)
**Length of stay <3 d (n = 8050)**		
Asian or Pacific Islander		
Model 1^b^	1.02 (0.84-1.23)	0.93 (0.77-1.13)
Model 2^c^	1.01 (0.84-1.23)	0.94 (0.78-1.15)
Model 3^d^	0.96 (0.78-1.17)	0.96 (0.79-1.18)
Black		
Model 1^b^	1.41 (1.2-1.66)	1.34 (1.14-1.59)
Model 2^c^	1.25 (1.05-1.49)	1.18 (0.99-1.41)
Model 3^d^	1.19 (1.00-1.43)	1.19 (0.99-1.43)
Hispanic		
Model 1^b^	1.08 (0.97-1.20)	1.02 (0.91-1.14)
Model 2^c^	0.90 (0.78-1.03)	0.92 (0.80-1.06)
Model 3^d^	0.88 (0.76-1.01)	0.96 (0.83-1.11)
**Length of stay ≥3 d (n = 10 596)**		
Asian or Pacific Islander		
Model 1^b^	0.89 (0.75-1.06)	1.04 (0.87-1.24)
Model 2^c^	0.89 (0.75-1.07)	1.05 (0.88-1.25)
Model 3^d^	0.88 (0.73-1.06)	1.08 (0.90-1.30)
Black		
Model 1^b^	1.38 (1.20-1.58)	1.28 (1.11-1.48)
Model 2^c^	1.21 (1.04-1.40)	1.18 (1.01-1.37)
Model 3^d^	1.15 (0.98-1.35)	1.20(1.02-1.41)
Hispanic		
Model 1^b^	1.17 (1.06-1.28)	0.92 (0.83-1.01)
Model 2^c^	1.03 (0.92-1.17)	0.88 (0.78-1.00)
Model 3^d^	1.03 (0.91-1.18)	0.93 (0.81-1.05)

Abbreviation: OR, odds ratio.

^a^ All models used White as a reference group as we assumed they would be least likely to experience stigmatizing language.

^b^ Model 1 includes race and ethnicity (unadjusted).

^c^ Model 2 includes race, ethnicity, and demographic characteristics (maternal age, marital status, insurance type, and language).

^d^ Model 3 includes race, ethnicity, demographic characteristics, and birth characteristics (parity, mode of birth, and gestational age).

Asian or Pacific Islanders were significantly less likely to have power or privilege language documented than White patients in model 3 (OR, 0.72; 95% CI, 0.58-0.91; *P* = .005) (Table 5). Black patients were more likely than White patients to have difficult patient language in model 2 (OR, 1.16; 95% CI, 1.03-1.31; *P* = .02); however, this association was no longer statistically significant after adjusting for birth characteristics. Black patients were also more likely to have language from the preferred language and/or respecting patient autonomy category after adjusting for demographics and birth characteristics (OR, 1.23; 95% CI, 1.09-1.39; *P* = .001). Hispanic patients were significantly less likely to have power and/or privilege language documented after adjustment for demographics and birth characteristics than White patients (OR, 0.62; 95% CI, 0.53-0.73; *P* < .001).

**Table 5.** Unadjusted and Adjusted Logistic Regression Models Examining Stigmatizing and Positive Language by Category and Race and Ethnicity

Language category	Odds ratio (95% CI)^a^					
	Marginalized language or identity	Difficult patient	Unilateral or authoritarian decisions	Questioning patient credibility^b^	Preferred language and/or patient autonomy	Power and/or privilege
**Asian or Pacific Islander (n = 1177)**						
Model 1^c^	0.74 (0.54-1.03)	0.99 (0.86-1.15)	0.96 (0.83-1.11)	NA	1.04 (0.92-1.19)	0.69 (0.56-0.85)
Model 2^d^	0.73 (0.53-1.02)	1.00 (0.86-1.15)	0.95 (0.82-1.11)	NA	1.05 (0.93-1.20)	0.71 (0.57-0.88)
Model 3^e^	0.76 (0.54-1.06)	0.99 (0.85-1.15)	0.92 (0.79-1.08)	NA	1.08 (0.95-1.24)	0.72 (0.58-0.91)
**Black (n = 2121)**						
Model 1^c^	2.48 (2.06-3.00)	1.23 (1.10-1.38)	1.12 (1.00-1.26)	1.01 (0.18-5.50)	1.35 (1.21-1.50)	1.02 (0.88-1.19)
Model 2	1.25 (1.01-1.55)	1.16 (1.03-1.31)	1.15 (1.01-1.30)	0.35 (0.05-2.33)	1.20 (1.07-1.35)	0.99 (0.84-1.18)
Model 3	1.26 (1.01-1.58)	1.11 (0.97-1.26)	1.08 (0.95-1.23)	NA	1.23 (1.09-1.38)	0.94 (0.79-1.12)
**Hispanic (n = 11 078)**						
Model 1^c^	2.17 (1.87-2.51)	0.96 (0.89-1.04)	0.93 (0.85-1.00)	0.96 (0.30-3.07)	1.07 (0.99-1.15)	0.50 (0.45-0.56)
Model 2	0.90 (0.74-1.09)	0.94 (0.85-1.04)	0.94 (0.85-1.04)	0.35 (0.08-1.59)	0.97 (0.88-1.06)	0.62 (0.53-0.72)
Model 3	0.94 (0.77-1.15)	0.95 (0.85-1.05)	0.92 (0.82-1.02)	NA	1.02 (0.93-1.13)	0.62 (0.53-0.73)

Abbreviations: NA, not applicable.

^a^ All models used White as a reference group as we assumed they would be least.

^b^ Due to the low occurrence of questioning patient credibility category, we were not able to provide some estimates.

^c^ Model 1 includes race and ethnicity (unadjusted).

^d^ Model 2 includes race, ethnicity, and demographic characteristics (maternal age, marital status, insurance type, and language).

^e^ Model 3 includes race, ethnicity, demographic characteristics, and birth characteristics (parity, mode of birth, and gestational age).

We also conducted additional analyses including selected clinical risk factors as covariates (model 4) (eTables 5 and 6 in Supplement 1). In fully adjusted models, Black patients were more likely than White patients to have preferred and/or respecting patient autonomy language (OR, 1.19; 95% CI, 1.05-1.34; *P* = .005), and Hispanic patients were less likely to have power and/or privilege language documented than White patients (OR, 0.78; 95% CI, 0.72-0.84; *P* < .001). Additional analyses, including length of stay subgroup analysis by language category (eFigure 1 in Supplement 1), goodness-of-fit of the logistic regression models (eTable 7 in Supplement 1), and any stigmatizing or positive language by race and ethnicity (eFigure 2 in Supplement 1).

## Discussion

The primary aim of this study was to examine the associations between patient race and ethnicity and the documentation of stigmatizing and positive language in birth admission clinical notes. We reviewed clinical notes to identify instances of stigmatizing and positive language using NLP, a text classification technique within large text datasets such as EHR. In particular, we conducted a context-level analysis approach to capture the nuances of language use in clinical documentation. Our findings indicate that Black patients had higher odds of having stigmatizing language documented in their clinical notes compared with White patients, particularly in the marginalized language or identities and difficult patient categories. Additionally, Hispanic had lower odds of positive language documented in their clinical notes compared with non-Hispanic White patients, particularly in the power and/or privilege category. Notably, as we included more covariates in the models, particularly in the fully adjusted model 4, we observed fewer statistically significant results. This is likely due to smaller sample sizes for specific language categories and racial and ethnic groups. Given that a similar study with a much larger sample found statistically significant associations,^32^ we aim to expand our future study with a larger sample size.

Our findings align with previous research highlighting racial and ethnic disparities in documentation practices in clinical settings. For example, Black patients are more likely to be described using stigmatizing language (eg, noncompliant, difficult, or resistant) in clinical notes among those who were treated in inpatient, outpatient, or emergency department settings.^19^ Similarly, we found that Black patients had the highest frequency of stigmatizing language documented in their clinical notes. This may be explained by implicit biases that influence clinician perceptions and documentation practices. These biases can affect a clinician’s decision-making for treatments and their interaction with patients, contributing to racial and ethnic disparities in documentation practices.^33^ For example, the higher odds of documentation of stigmatizing language in the difficult patient category could suggest that clinicians may perceive Black patients as challenging to manage, a notion that has been historically perpetuated in clinical settings.^34^

We also found that Black patients were more likely to have positive language documented, particularly in the preferred and/or autonomy category. This duality may indicate a more complex interplay between factors that influence language use and clinical documentation in clinical settings. One possible explanation is that clinicians may show “overt positive behaviors or expressions” to counterbalance potential implicit bias,^35^ a concept known as overcorrection in psychology.^36^ However, there is no direct evidence supporting this in health care settings. Given the limited literature on the documentation of positive language in clinical notes, particularly in birth settings, future research is needed to further examine the complex relationships between positive language in clinical notes and race and ethnicity. Previous research found that Hispanic patients with diabetes were less likely to have positive language documented.^22^ This was consistent with our study findings for Hispanic patients. Limited use of positive language may indicate the undervaluing of a patient’s autonomy and agency in clinical settings among Hispanic patients.

Importantly, identifying stigmatizing and positive language in clinical notes is a developing area of research that is subjective in nature. We performed rigorous qualitative analysis, informed by prior research, to enhance the reliability of our language categorizations. Our multidisciplinary team with different cultural and professional backgrounds, including physicians, nurses, and data scientists, provided diverse perspectives to improve the rigor and trustworthiness of our qualitative work. However, language that some may perceive as stigmatizing might also be seen as neutral or objective description to others. For example, “FOB [father of the baby] works for [a prominent company]” may be regarded as an objective statement about patient’s family. However, we observed that such descriptions were not consistently documented for all patients, and not necessarily relevant to patient care. When such details are selectively included, they may unintentionally highlight or imply a socioeconomic status of patients, potentially leading to bias. Additionally, we acknowledge that certain language commonly used in clinical documentation, such as “refused any intervention,” may not always carry negative connotations. We did not aim to discern the true intent of writers’ language use in the current study, but examined the documentation patterns and how they may differ by patient race and ethnicity. Future research will greatly benefit this field by incorporating clinician perspectives via qualitative methods and characteristics (eg, years in practice, health care role, note types) to examine the motivation or factors influencing certain language use in clinical documentation.

Recognizing that these notes were taken from a prepandemic period is important. Cultural humility and antiracism training have increased since the COVID-19 pandemic and may not reflect current practices or clinician beliefs.^37^ Furthermore, though diversity, equity and inclusion training have been ongoing in many clinical settings to reduce racism and bias in health care, few programs have been evaluated for their effects on clinical documentations and patient outcomes.^38^ Evaluation of antiracism educational programs is critical as previous studies have shown that clinicians hold discriminatory beliefs against Black patients in labor and birth settings as a reflection of US society and the legacy of slavery,^39,40,41^ and that these biases are associated with adverse health outcomes.^5,42,43^

Notably, our study employed novel and advanced NLP methods, extending the work of previous studies that focused on word-level analysis or anchor word approaches. While these methods have provided valuable insights, they are limited in their ability to capture the full complexity and context-dependent nature of language use in clinical documentation. Our context-level analysis used advanced NLP methods and offered a more comprehensive examination of language patterns in labor and birth clinical notes. This approach allowed us to detect nuances that other methods might miss. For instance, our method can identify potential implicit bias in demographic descriptions that might seem neutral at the word level, such as “patient is a 27 [year old] African American single unemployed female,” where the combination of descriptors could subtly reinforce marginalized identities.

Together, these findings have important implications for clinical practice. Findings of this study can be used to inform tailored education, training, and policy review to identify stigmatizing language use and to address potential bias in clinical documentation and communication. Clinician education should focus on patient-centered language to reduce stigmatizing language and promote equitable documentation practices. However, simply removing language from documentation will not eliminate potential underlying biases, and the integration of bias-free templates and prompts in EHR to standardize documentation should be implemented with caution. Efforts to implement software that flags stigmatizing language in real time, for example, should be paired with training to understand why it is being used. This technology could support education efforts to interrupt bias at the time of documentation with the intention of changing behaviors. Potential barriers such as clinician resistance and workflow integration should be considered in the implementation of such education and technology to proactively address concerns related to feelings of surveillance of clinical notes and reduced autonomy in documentation practices. Adopting a user-centered approach and design to technology implementation should incorporate clinician and other stakeholder feedback to ensure data security while addressing the primary intended goal of improving patient care and reducing stigmatizing language that may contribute to disparities. Implementation of such techniques may introduce potential challenges, including clinician resistance and workflow integration ethnical consideration related to surveillance of clinical notes and autonomy in documentation practices.^44^ Adopting a user-centered approach and design to incorporate clinician and other stakeholder feedback and ensuring data security would be critical to overcome these challenges.^45^

### Limitations

This study has several limitations. Our study was conducted in a single health care system with a high proportion of Hispanic and Medicaid-insured patients which may limit generalizability to other settings. As is common in many studies using EHR data, we had significant amounts of missing data for race and ethnicity. Although we mitigated this limitation by using the highly accurate BISFG race and ethnicity imputation method, we acknowledge that self-reported race and ethnicity is the criterion standard. Future studies should be conducted with other patient populations and include a more refined categorization of race and ethnicity to disentangle findings. For example, there may be a difference in the amount of stigmatizing language documented for Hispanic Black patients compared with non-Hispanic Black patients. Including information about other marginalized identities, such as English proficiency levels, may also be important in future analyses.

In addition, while we adjusted for key demographic covariates, other important factors that may also influence documentation patterns were unavailable in the current study. For example, we did not have hospital-level data to account for potential clustering effects, preventing us from determining which of each of the 2 hospitals included in the study the note originated from. While we expect minimal variability given that both hospitals are in the same geographic area and part of the same hospital system, it could still introduce potential clustering effects. We also did not have clinician-level data. Primary data collection of clinician characteristics such as level of training, discipline, age, and gender may allow for a better understanding of where to focus educational interventions.

There were also other patient-level factors that may have influenced stigmatizing language documentation, including access to prenatal care, contraceptive plan, and substance or smoking use that were unavailable in this dataset and warrant examination in future studies. Furthermore, study data were collected before the COVID-19 pandemic. Although perinatal health inequities have held steady or worsened compared to the pre-COVID-19 era, additional studies are needed to examine the potential changes in documentation practices and language use in the post-COVID-19 context.

We acknowledge an additional limitation relating to our definition and conceptualization of stigmatizing language. These were based on qualitative work that may reflect our own biases and subjectivity despite our efforts to enhance the rigor and trustworthiness (eg, multidisciplinary team, conceptual foundation informed by previous literature, iterative qualitative coding process). Future studies should validate and refine these categories using additional approaches, such as patient perspectives or clinician feedback to enhance the validity and expand the generalizability of our findings. In addition, some of our findings were no longer statistically significant after adjustment for clinical characteristics. This may have been due to smaller available sample sizes for some of the language categories and racial and ethnic subgroups. Our fully adjusted model (model 4) also had a poorer fit based on a significant Hosmer-Lemeshow *P* value, suggesting the need to further refine the model. Lastly, although our NLP model achieved good performance in previous studies to detect stigmatizing and positive language (overall F1 score, precision, and recall at 0.8), there is always a potential chance of misclassification, including missed instances of stigmatizing and positive language (false negatives) and inaccurate identification of cases (false positives).

## Conclusion

We used an advanced NLP approach to analyze the use of stigmatizing and positive language in labor and birth clinical notes by patient race and ethnicity. Black patients were more likely to have both stigmatizing and positive language documented, while Hispanic patients experienced lower odds of positive language documentation. These findings underscore the importance of implementing targeted interventions to mitigate biases in perinatal care and to foster documentation practices that are both equitable and culturally sensitive. Future research should focus on developing an NLP system that can pick up on the nuances of these relationships to automate the detection of stigmatizing language and provide alternatives or alerts to interrupt bias, reduce bias in documentation, and contribute to achieving perinatal health equity.

## Notes

Supplement 1. **eTable 1.** All Note Types Containing Free-Text Narratives **eTable 2.** *P* Values From χ^2^ Tests Between Main Study Variables and Covariates **eTable 3.** Multicollinearity Assessment, Correlation Matrix for Race, Ethnicity, and Other Covariates **eTable 4.** Multicollinearity Assessment, Variance Inflation Factors (VIF) for Primary Study Variables and Covariates **eTable 5.** Fully Adjusted Multivariable Regression Model for Full Sample (Model 4), Including Race and Ethnicity, Demographics, Birth Characteristics, and Clinical Comorbidities **eTable 6.** Fully Adjusted Multivariable Regression Model (Model 4)a by Length of Stay Subgroup (Median Split at 3 Days) **eTable 7.** Logistic Regression Model Fit **eFigure 1.** Unadjusted and Adjusted Logistic Regression Models Examining Stigmatizing and Positive Language and Patient Race and Ethnicity, by Length of Stay Subgroup (Median Split at 3 Days) **eFigure 2.** Unadjusted and Adjusted Logistic Regression Models Examining Any Stigmatizing or Positive Language by Patient Race and Ethnicity in the Full Study Sample

Supplement 2.
Data Sharing Statement
