# Supplementary material for: Stigmatizing and Positive Language in Birth Clinical Notes Associated With Race and Ethnicity
Source: JAMA Netw Open. 2025 May 13;8(5):e259599. doi: 10.1001/jamanetworkopen.2025.9599 (PMC12076172; doi:10.1001/jamanetworkopen.2025.9599)
Supplement: Supplement 1. — eTable 1. All Note Types Containing Free-Text Narratives eTable 2. P Values From χ2 Tests Between Main Study Variables and Covariates eTable 3. Multicollinearity Assessment, Correlation Matrix for Race, Ethnicity, and Other Covariates eTable 4. Multicollinearity Assessment, Variance Inflation Factors (VIF) for Primary Study Variables and Covariates eTable 5. Fully Adjusted Multivariable Regression Model for Full Sample (Model 4), Including Race and Ethnicity, Demographics, Birth Characteristics, and Clinical Comorbidities eTable 6. Fully Adjusted Multivariable Regression Model (Model 4)a by Length of Stay Subgroup (Median Split at 3 Days) eTable 7. Logistic Regression Model Fit eFigure 1. Unadjusted and Adjusted Logistic Regression Models Examining Stigmatizing and Positive Language and Patient Race and Ethnicity, by Length of Stay Subgroup (Median Split at 3 Days) eFigure 2. Unadjusted and Adjusted Logistic Regression Models Examining Any Stigmatizing or Positive Language by Patient Race and Ethnicity in the Full Study Sample [file jamanetwopen-e259599-s001.pdf]

# Supplemental Online Content

Hulchafo II, Scroggins JK, Harkins SE, et al. Stigmatizing and positive language in birth clinical notes associated with race and ethnicity. *JAMA Netw Open.* 2025;8(5):e259599. doi:10.1001/jamanetworkopen.2025.9599

**eTable 1.** All Note Types Containing Free-Text Narratives

**eTable 2.** *P* Values From $\chi^2$ Tests Between Main Study Variables and Covariates

**eTable 3.** Multicollinearity Assessment, Correlation Matrix for Race, Ethnicity, and Other Covariates

**eTable 4.** Multicollinearity Assessment, Variance Inflation Factors (VIF) for Primary Study Variables and Covariates

**eTable 5.** Fully Adjusted Multivariable Regression Model for Full Sample (Model 4), Including Race and Ethnicity, Demographics, Birth Characteristics, and Clinical Comorbidities

**eTable 6.** Fully Adjusted Multivariable Regression Model (Model 4)a by Length of Stay Subgroup (Median Split at 3 Days)

**eTable 7.** Logistic Regression Model Fit

**eFigure 1.** Unadjusted and Adjusted Logistic Regression Models Examining Stigmatizing and Positive Language and Patient Race and Ethnicity, by Length of Stay Subgroup (Median Split at 3 Days)

**eFigure 2.** Unadjusted and Adjusted Logistic Regression Models Examining Any Stigmatizing or Positive Language by Patient Race and Ethnicity in the Full Study Sample

This supplemental material has been provided by the authors to give readers additional information about their work.

© 2025 Hulchafo II et al. *JAMA Network Open*.

**eTable 1.** All Note Types Containing Free-Text Narratives

| Note Types |
| --- |
| Miscellaneous Nursing Note |
| Obstetric Labor Progress Note |
| Obstetric Postpartum Note |
| NewYork-Presbyterian Discharge Summary Note |
| Obstetric Postoperative Note |
| Obstetric Delivery Note |
| Obstetric Antepartum Progress Note |
| Obstetric/Gynecology Encounter Note |
| Obstetric Triage Note |
| Social Work/Case Management Initial Comprehensive Assessment |
| Obstetric Admission Note |
| Obstetric Attending/Fellow Note |
| Social Work/Case Management Progress/Reassessment Note |
| Social Work/Case Management Discharge Planning/Final Note |
| Obstetric Preoperative Note |
| Nutrition Note |
| Social Work Consult Note |
| Anesthesia Resident Note |
| Nutrition Screen  Obstetric |
| Initial Nutrition Assessment |
| Attending Note |
| Neonatal Fellow (NICU) Note |

© 2025 Hulchafo II et al. *JAMA Network Open*.

**eTable 2.** P-values from χ² Tests Between Main Study Variables and Covariates

| | Race/ ethnicity | Any Stigmatizing language | Marginalized language/ identities | Difficult patient | Unilateral /authoritarian decisions | Questioning patient credibility | Any positive language | Preferred language/respecting patient autonomy | Power/ privilege | Any stigmatizing or positive language |
|---|---|---|---|---|---|---|---|---|---|---|
| Age | <0.001 | <0.001 | <0.001 | 0.04 | 0.001 | 0.08 | <0.001 | <0.001 | <0.001 | <0.001 |
| Insurance | <0.001 | <0.001 | <0.001 | 0.24 | 0.69 | 0.08 | 0.02 | <0.001 | <0.001 | <0.001 |
| Pre-eclampsia | <0.001 | <0.001 | 0.14 | <0.001 | <0.001 | 1.00 | <0.001 | <0.001 | <0.001 | <0.001 |
| Gestational diabetes | <0.001 | 0.03 | 0.11 | <0.001 | 0.003 | 0.67 | 0.02 | 0.02 | 0.09 | 0.04 |
| BMI ≥ 30 | <0.001 | <0.001 | <0.001 | 0.03 | 0.008 | 0.69 | 0.004 | 0.01 | 0.001 | 0.009 |
| Marital status | <0.001 | <0.001 | <0.001 | 0.13 | 0.003 | 0.69 | 0.002 | <0.001 | 0.60 | <0.001 |
| Parity | <0.001 | 0.002 | <0.001 | 0.001 | 0.78 | 0.97 | <0.001 | <0.001 | 0.005 | <0.001 |
| Gestational age | <0.001 | <0.001 | 0.41 | <0.001 | <0.001 | 0.14 | <0.001 | <0.001 | <0.001 | <0.001 |
| Mode of birth | <0.001 | <0.001 | 0.002 | <0.001 | <0.001 | 1.00 | <0.001 | <0.001 | <0.001 | 0.001 |
| Language | <0.001 | 0.99 | <0.001 | 0.001 | 0.63 | 0.60 | <0.001 | 0.004 | <0.001 | 0.001 |
| Length of stay | 0.091 | 0.34 | 0.26 | 0.29 | 0.02 | 0.22 | 0.44 | 0.65 | 0.53 | 0.25 |

© 2025 Hulchafo II et al. *JAMA Network Open*.

**eTable 3.** Multicollinearity Assessment, Correlation Matrix for Race, Ethnicity, and Other Covariates

| | Pre-eclampsia | Gestational diabetes | BMI ≥ 30 | Age | Gestational age | parity | insurance | Marital Status | Race/ Ethnicity | Language | Mode of birth | Length of stay |
|---|---|---|---|---|---|---|---|---|---|---|---|---|
| Pre-eclampsia | 1.00 | 0.04 | 0.07 | 0.02 | -0.19 | 0.08 | -0.01 | 0.03 | -0.03 | -0.01 | 0.09 | -0.00 |
| Gestational diabetes | | 1.00 | 0.10 | 0.09 | -0.05 | -0.04 | -0.00 | -0.02 | -0.03 | 0.02 | 0.06 | 0.01 |
| BMI ≥ 30 | | | 1.00 | -0.02 | -0.02 | -0.04 | -0.09 | 0.09 | -0.07 | 0.01 | 0.08 | 0.01 |
| Age | | | | 1.00 | -0.06 | -0.13 | 0.22 | -0.19 | 0.07 | -0.08 | 0.11 | 0.00 |
| Gestational age | | | | | 1.00 | 0.01 | -0.03 | 0.02 | -0.00 | 0.04 | -0.15 | 0.00 |
| parity | | | | | | 1.00 | 0.13 | 0.01 | 0.03 | -0.10 | -0.03 | -0.00 |
| insurance | | | | | | | 1.00 | 0.47 | 0.26 | 0.41 | 0.00 | 0.00 |
| Marital Status | | | | | | | | 1.00 | 0.25 | 0.19 | 0.02 | -0.01 |
| Race/ Ethnicity | | | | | | | | | 1.00 | 0.08 | -0.03 | -0.01 |
| Language | | | | | | | | | | 1.00 | -0.04 | -0.00 |
| Mode of birth | | | | | | | | | | | 1.00 | -0.00 |
| Length of stay | | | | | | | | | | | | 1.00 |

© 2025 Hulchafo II et al. *JAMA Network Open*.

**eTable 4.** Multicollinearity Assessment, Variance Inflation Factors (VIF) for Primary Study Variables and Covariates

| Independent Variables | VIF |
|---|---|
| Pre-eclampsia | 1.06 |
| Gestational diabetes | 1.03 |
| BMI ≥ 30 | 1.05 |
| Maternal age group <20 | 1.07 |
| Maternal age group >35 | 1.18 |
| Marital status divorced | 1.02 |
| Marital status other | 1.03 |
| Marital status single | 1.57 |
| Marital status widowed | 1.00 |
| Language non-English | 1.36 |
| Race/Ethnicity Asian Pacific Islander | 1.20 |
| Race/Ethnicity Black | 1.55 |
| Race/Ethnicity Hispanic | 2.50 |
| Parity nulliparous | 1.12 |
| Gestational age preterm | 1.06 |
| Insurance Medicaid | 1.87 |
| Mode of birth c-section | 1.06 |
| Length of stay above median | 1.00 |

© 2025 Hulchafo II et al. *JAMA Network Open*.

**eTable 5.** Fully Adjusted Multivariable Regression Model for Full Sample (Model 4)[a], Including Race and Ethnicity, Demographics, Birth Characteristics and Clinical Comorbidities (N=18,646)

| | **Asian/Pacific Islander (n=1,177)** | | **Black (n=2,121)** | | **Hispanic (n=11,078)** | | **White (n=4,270)** |
|---|---|---|---|---|---|---|---|
| **Language Category** | **OR (95% CI)** | ***P* value** | **OR (95% CI)** | ***P* value** | **OR (95% CI)** | ***P* value** | |
| **Any Stigmatizing Language** | 0.92 (0.80 - 1.06) | 0.24 | 1.12 (0.99 - 1.26) | 0.06 | 0.93 (0.84 - 1.03) | 0.15 | Ref. |
| Marginalized language/identities | 0.77 (0.55 - 1.08) | 0.13 | 1.21 (0.97 - 1.51) | 0.10 | 0.90 (0.74 - 1.10) | 0.32 | Ref. |
| Difficult patient | 0.99 (0.85 - 1.15) | 0.87 | 1.06 (0.93 - 1.20) | 0.41 | 0.91 (0.82 - 1.02) | 0.10 | Ref. |
| Unilateral/authoritarian Decisions | 0.92 (0.79 - 1.07) | 0.30 | 1.05 (0.93 - 1.20) | 0.42 | 0.90 (0.81 - 1.00) | 0.06 | Ref. |
| Questioning patient Credibility | --[b] | | --[b] | | --[b] | | Ref. |
| **Any Positive Language** | 1.03 (0.90 - 1.18) | 0.71 | 1.15 (1.02 - 1.30) | 0.02 | 0.91 (0.83 - 1.01) | 0.07 | Ref. |
| Preferred language/ respecting patient autonomy | 1.08 (0.95 - 1.24) | 0.24 | 1.19 (1.05 - 1.34) | 0.005 | 0.99 (0.90 - 1.10) | 0.92 | Ref. |
| Power/privilege | 0.93 (0.88 - 0.98) | 0.006 | 0.97 (0.92 - 1.02) | 0.27 | 0.78 (0.72 - 0.84) | <0.001 | Ref. |
| **Any Stigmatizing or Positive Language** | 0.99 (0.85 - 1.15) | 0.92 | 1.26 (1.09 - 1.45) | 0.001 | 0.97 (0.87 - 1.08) | 0.61 | Ref. |

[a] Model 4: Fully adjusted model included race and ethnicity, demographic characteristics (maternal age, marital status, insurance type, language), birth characteristics (parity, mode of birth, gestational age), and clinical risk factors (preeclampsia, gestational diabetes, body mass index ≥ 30).

[b] Due to the low occurrence of questioning patient credibility category, we were not able to provide estimates.

Abbreviation: OR, Odds Ratio; CI, Confidence Intervals.

© 2025 Hulchafo II et al. *JAMA Network Open*.

**eTable 6.** Fully Adjusted Multivariable Regression Model (Model 4)[a] by Length of Stay Subgroup (Median Split at 3 Days)

### A. Length of Stay < 3 days (n=8,050)

| | Asian/Pacific Islander | | Black | | Hispanic | | White |
|---|---|---|---|---|---|---|---|
| | (n= 545) | | (n= 885) | | (n= 4,756) | | (n= 1,864) |
| **Language Category** | **OR (95% CI)** | ***P* value** | **OR (95% CI)** | ***P* value** | **OR (95% CI)** | ***P* value** | |
| **Any Stigmatizing Language** | 0.96 (0.79 - 1.17) | 0.69 | 1.12 (0.93 - 1.34) | 0.22 | 0.84 (0.72 - 0.97) | 0.02 | Ref. |
| Marginalized language/identities | 0.69 (0.41 - 1.14) | 0.14 | 1.33 (0.96 - 1.84) | 0.09 | 0.87 (0.65 - 1.17) | 0.36 | Ref. |
| Difficult patient | 1.02 (0.81 - 1.27) | 0.87 | 0.94 (0.77 - 1.15) | 0.57 | 0.83 (0.71 - 0.98) | 0.03 | Ref. |
| Unilateral/authoritarian Decisions | 0.99 (0.80 - 1.24) | 0.95 | 1.08 (0.89 - 1.32) | 0.44 | 0.85 (0.72 - 1.00) | 0.05 | Ref. |
| Questioning patient Credibility | --[b] | | --[b] | | --[b] | | Ref. |
| **Any Positive Language** | 0.97 (0.79 - 1.18) | 0.74 | 1.13 (0.94 - 1.36) | 0.18 | 0.93 (0.80 - 1.08) | 0.36 | Ref. |
| Preferred language/ respecting patient autonomy | 1.08 (0.88 - 1.31) | 0.47 | 1.18 (0.98 - 1.42) | 0.07 | 1.05 (0.91 - 1.22) | 0.50 | Ref. |
| Power/privilege | 0.72 (0.52 - 1.01) | 0.06 | 0.87 (0.66 - 1.13) | 0.29 | 0.59 (0.46 - 0.74) | <0.001 | Ref. |
| **Any Stigmatizing or Positive Language** | 0.95 (0.76 - 1.19) | 0.65 | 1.25 (1.00 - 1.55) | 0.05 | 0.92 (0.78 - 1.09) | 0.33 | Ref. |

### B. Length of Stay ≥ 3 days (n=10,596)

| | Asian/Pacific Islander | | Black | | Hispanic | | White |
|---|---|---|---|---|---|---|---|
| | (n= 632) | | (n= 1,236) | | (n= 6,322) | | (n= 2,406) |
| **Language Category** | **OR (95% CI)** | ***P* value** | **OR (95% CI)** | ***P* value** | **OR (95% CI)** | ***P* value** | |
| **Any Stigmatizing Language** | 0.88 (0.73 - 1.06) | 0.19 | 1.13 (0.96 - 1.32) | 0.13 | 1.01 (0.89 - 1.16) | 0.82 | Ref. |
| Marginalized language/identities | 0.84 (0.53 - 1.32) | 0.44 | 1.12 (0.83 - 1.52) | 0.45 | 0.93 (0.71 - 1.22) | 0.60 | Ref. |
| Difficult patient | 0.96 (0.78 - 1.18) | 0.70 | 1.15 (0.96 - 1.36) | 0.12 | 0.98 (0.85 - 1.13) | 0.77 | Ref. |
| Unilateral/authoritarian Decisions | 0.85 (0.69 - 1.06) | 0.15 | 1.04 (0.87 - 1.24) | 0.64 | 0.95 (0.82 - 1.10) | 0.49 | Ref. |

© 2025 Hulchafo II et al. *JAMA Network Open*.

| | | | | | | | |
|---|---|---|---|---|---|---|---|
| Questioning patient Credibility | --[b] | | --[b] | | --[b] | | Ref. |
| **Any Positive Language** | 1.08 (0.90 - 1.30) | 0.42 | 1.17 (0.99 - 1.37) | 0.06 | 0.90 (0.79 - 1.02) | 0.11 | Ref. |
| Preferred language/ respecting patient autonomy | 1.09 (0.91 - 1.31) | 0.34 | 1.19 (1.01 - 1.39) | 0.04 | 0.95 (0.84 - 1.08) | 0.46 | Ref. |
| Power/privilege | 0.74 (0.54 - 1.00) | 0.05 | 0.95 (0.75 - 1.20) | 0.65 | 0.61 (0.49 - 0.76) | <0.001 | Ref. |
| **Any Stigmatizing or Positive Language** | 1.02 (0.83 - 1.26) | 0.83 | 1.28 (1.06 - 1.54) | 0.009 | 1.02 (0.88 - 1.18) | 0.81 | Ref. |

[a] Model 4: Fully adjusted model included race and ethnicity, demographic characteristics (maternal age, marital status, insurance type, language), birth characteristics (parity, mode of birth, gestational age), and clinical risk factors (preeclampsia, gestational diabetes, body mass index ≥ 30).

[a] Due to the low occurrence of questioning patient credibility category, we were not able to provide estimates.

Abbreviation: OR, Odds Ratio; CI, Confidence Intervals.

© 2025 Hulchafo II et al. *JAMA Network Open*.

**eTable 7.** Logistic Regression Model Fit

| Model | Outcome Variables | Hosmer- Lemeshow | | Mean Pearson Residual |
|---|---|---|---|---|
| | | statistic | *P* Value | |
| Model 1 | Any stigmatizing language | 0.00 | 1.00 | 0.000 |
| | Marginalized language/identities | 0.00 | 1.00 | 0.000 |
| | Difficult patient | 0.00 | 1.00 | 0.000 |
| | Unilateral/authoritarian decisions | 0.00 | 1.00 | 0.000 |
| | Questioning patient credibility | 0.00 | 1.00 | 0.000 |
| | Any positive language | 0.00 | 1.00 | 0.000 |
| | Preferred language/respecting patient autonomy | 0.00 | 1.00 | 0.000 |
| | Power/privilege | 0.00 | 1.00 | 0.000 |
| | Any Stigmatizing or Positive Language | 0.00 | 1.00 | 0.000 |
| Model 2 | Any stigmatizing language | 9.28 | 0.32 | 0.000 |
| | Marginalized language/identities | 24.71 | 0.002 | 0.000 |
| | Difficult patient | 13.02 | 0.11 | 0.000 |
| | Unilateral/authoritarian decisions | 7.10 | 0.53 | 0.000 |
| | Questioning patient credibility | 21.91 | 0.005 | 0.002 |
| | Any positive language | 8.68 | 0.37 | 0.000 |
| | Preferred language/respecting patient autonomy | 11.70 | 0.17 | 0.000 |
| | Power/privilege | 11.97 | 0.15 | 0.000 |
| | Any Stigmatizing or Positive Language | 9.52 | 0.30 | 0.000 |
| Model 3 | Any stigmatizing language | 18.17 | 0.02 | 0.000 |
| | Marginalized language/identities | 12.29 | 0.14 | 0.000 |
| | Difficult patient | 11.46 | 0.18 | 0.000 |
| | Unilateral/authoritarian decisions | 17.28 | 0.03 | 0.000 |
| | Questioning patient credibility | -- | -- | -- |
| | Any positive language | 30.52 | 0 | -0.001 |
| | Preferred language/respecting patient autonomy | 12.76 | 0.12 | 0.000 |
| | Power/privilege | 9.23 | 0.32 | -0.001 |
| | Any Stigmatizing or Positive Language | 14.07 | 0.08 | 0.000 |

© 2025 Hulchafo II et al. *JAMA Network Open*.

| | | | | |
|---|---|---|---|---|
| | Any stigmatizing language | 13.34 | 0.10 | 0.000 |
| | Marginalized language/identities | 18.64 | 0.02 | -0.001 |
| | Difficult patient | 10.59 | 0.23 | 0.000 |
| | Unilateral/authoritarian decisions | 15.14 | 0.06 | 0.000 |
| Model 4 | Questioning patient credibility | --[a] | --[a] | --[a] |
| | Any positive language | 33.23 | <0.001 | -0.001 |
| | Preferred language/respecting patient autonomy | 14.87 | 0.06 | 0.000 |
| | Power/privilege | 10.60 | 0.23 | -0.002 |
| | Any Stigmatizing or Positive Language | 9.52 | 0.30 | 0.000 |

[a] Not able to estimate associations for the questioning patient credibility category due to insufficient sample size.

© 2025 Hulchafo II et al. *JAMA Network Open*.

**eFigure 1.** Unadjusted and Adjusted Logistic Regression Models Examining Stigmatizing and Positive Language and Patient Race and Ethnicity, by Length of Stay Subgroup (Median Split at 3 Days)

## A. Length of Stay < 3 days

### A-1. Asian/Pacific Islander (n=545)

| Language Category | Odds Ratio (95% Confidence Interval) | P value |
|---|---|---|
| **Marginalized language/ identities** | | |
| Model 1 | 0.71 (0.44 - 1.16) | 0.17 |
| Model 2 | 0.69 (0.42 - 1.12) | 0.13 |
| Model 3 | 0.67 (0.41 - 1.12) | 0.13 |

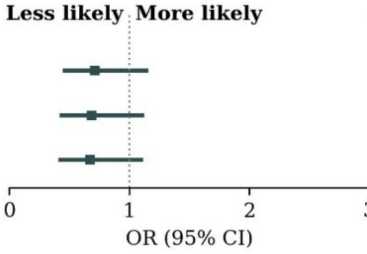


| Language Category | Odds Ratio (95% Confidence Interval) | P value |
|---|---|---|
| **Difficult patient** | | |
| Model 1 | 1.04 (0.84 - 1.28) | 0.74 |
| Model 2 | 1.04 (0.84 - 1.28) | 0.74 |
| Model 3 | 1.02 (0.81 - 1.27) | 0.89 |

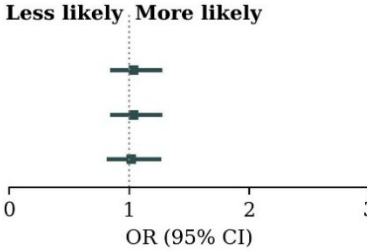


| Language Category | Odds Ratio (95% Confidence Interval) | P value |
|---|---|---|
| **Unilateral/authoritarian decisions** | | |
| Model 1 | 1.03 (0.84 - 1.28) | 0.75 |
| Model 2 | 1.03 (0.84 - 1.28) | 0.76 |
| Model 3 | 1.00 (0.80 - 1.24) | 0.98 |

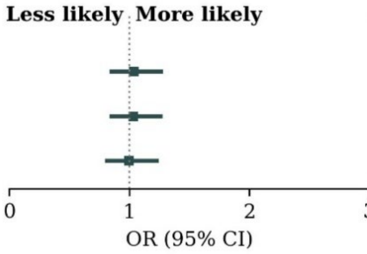


| Language Category | Odds Ratio (95% Confidence Interval) | P value |
|---|---|---|
| **Questioning patient credibility** | -- | |
| Model 1 | -- | |
| Model 2 | -- | |
| Model 3 | -- | |

| Language Category | Odds Ratio (95% Confidence Interval) | P value |
|---|---|---|
| **Preferred language/ respecting patient autonomy** | | |
| Model 1 | 1.03 (0.85 - 1.25) | 0.75 |
| Model 2 | 1.04 (0.86 - 1.26) | 0.67 |
| Model 3 | 1.07 (0.88 - 1.31) | 0.49 |

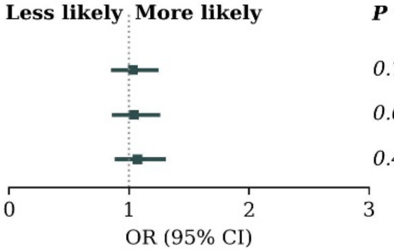


| Language Category | Odds Ratio (95% Confidence Interval) | P value |
|---|---|---|
| **Power/privilege** | | |
| Model 1 | 0.70 (0.51 - 0.96) | 0.03 |
| Model 2 | 0.73 (0.53 - 1.00) | 0.05 |
| Model 3 | 0.72 (0.52 - 1.00) | 0.05 |

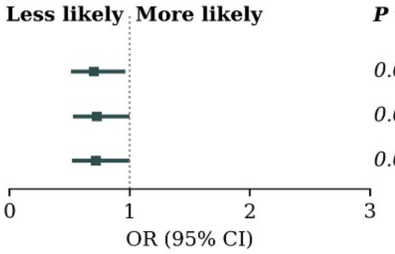


© 2025 Hulchafo II et al. *JAMA Network Open*.

## A-2. Black (n=885)

| Language Category | Odds Ratio (95% Confidence Interval) | P value |
|---|---|---|
| **Marginalized language/ identities** | | |
| Model 1 | 2.64 (1.99 - 3.50) | <0.001 |
| Model 2 | 1.38 (1.00 - 1.90) | 0.05 |
| Model 3 | 1.39 (1.01 - 1.93) | 0.05 |

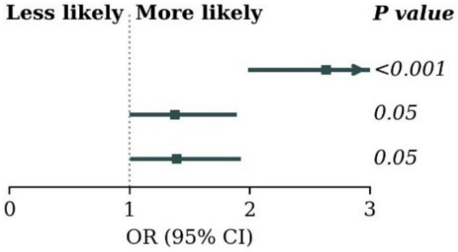


| Language Category | Odds Ratio (95% Confidence Interval) | P value |
|---|---|---|
| **Difficult patient** | | |
| Model 1 | 1.13 (0.95 - 1.35) | 0.16 |
| Model 2 | 1.05 (0.87 - 1.27) | 0.59 |
| Model 3 | 1.02 (0.84 - 1.24) | 0.84 |

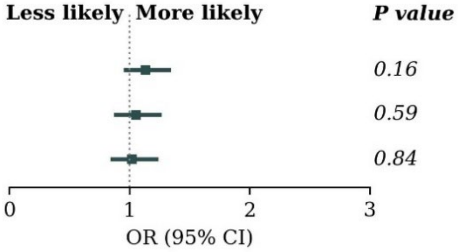


| Language Category | Odds Ratio (95% Confidence Interval) | P value |
|---|---|---|
| **Unilateral/authoritarian decisions** | | |
| Model 1 | 1.17 (0.98 - 1.39) | 0.08 |
| Model 2 | 1.22 (1.01 - 1.47) | 0.04 |
| Model 3 | 1.13 (0.93 - 1.38) | 0.21 |

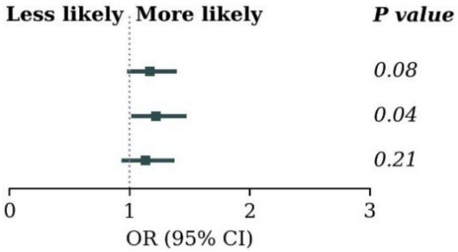


| Language Category | Odds Ratio (95% Confidence Interval) | |
|---|---|---|
| **Questioning patient credibility** | | -- |
| Model 1 | -- | |
| Model 2 | -- | |
| Model 3 | -- | |

| Language Category | Odds Ratio (95% Confidence Interval) | P value |
|---|---|---|
| **Preferred language/ respecting patient autonomy** | | |
| Model 1 | 1.39 (1.18 - 1.64) | <0.001 |
| Model 2 | 1.21 (1.02 - 1.44) | 0.03 |
| Model 3 | 1.24 (1.03 - 1.48) | 0.02 |

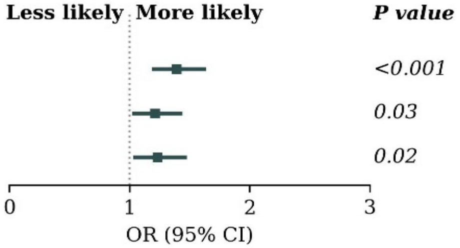


| Language Category | Odds Ratio (95% Confidence Interval) | P value |
|---|---|---|
| **Power/privilege** | | |
| Model 1 | 1.07 (0.84 - 1.35) | 0.59 |
| Model 2 | 0.99 (0.76 - 1.27) | 0.92 |
| Model 3 | 0.91 (0.69 - 1.18) | 0.46 |

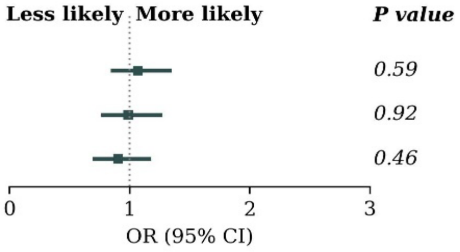


© 2025 Hulchafo II et al. *JAMA Network Open*.

## A-3. Hispanic (n=4,756)

| Language Category | Odds Ratio (95% Confidence Interval) | P value |
| --- | --- | --- |
| **Marginalized language/ identities** | | |
| Model 1 | 2.17 (1.73 - 2.70) | <0.001 |
| Model 2 | 0.91 (0.68 - 1.22) | 0.54 |
| Model 3 | 0.91 (0.68 - 1.22) | 0.52 |

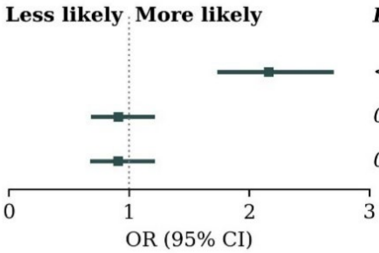


| Language Category | Odds Ratio (95% Confidence Interval) | P value |
| --- | --- | --- |
| **Difficult patient** | | |
| Model 1 | 0.91 (0.81 - 1.03) | 0.13 |
| Model 2 | 0.86 (0.74 - 1.00) | 0.06 |
| Model 3 | 0.88 (0.75 - 1.03) | 0.12 |

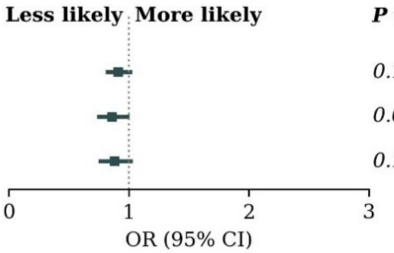


| Language Category | Odds Ratio (95% Confidence Interval) | P value |
| --- | --- | --- |
| **Unilateral/ authoritarian decisions** | | |
| Model 1 | 0.89 (0.79 - 1.00) | 0.05 |
| Model 2 | 0.91 (0.78 - 1.07) | 0.25 |
| Model 3 | 0.88 (0.74 - 1.03) | 0.11 |

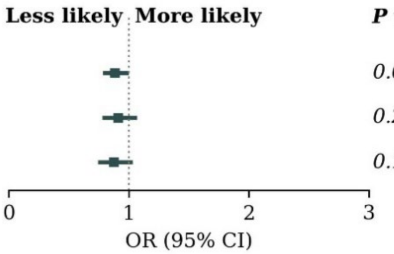


| Language Category | Odds Ratio (95% Confidence Interval) |
| --- | --- |
| **Questioning patient credibility** | -- |
| Model 1 | -- |
| Model 2 | -- |
| Model 3 | -- |

| Language Category | Odds Ratio (95% Confidence Interval) | P value |
| --- | --- | --- |
| **Preferred language/ respecting patient autonomy** | | |
| Model 1 | 1.16 (1.05 - 1.30) | 0.006 |
| Model 2 | 1.02 (0.89 - 1.18) | 0.76 |
| Model 3 | 1.08 (0.94 - 1.25) | 0.28 |

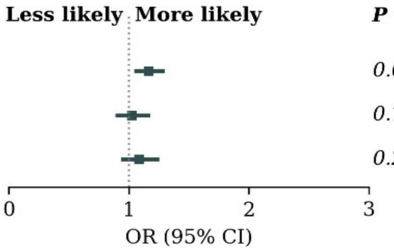


| Language Category | Odds Ratio (95% Confidence Interval) | P value |
| --- | --- | --- |
| **Power/privilege** | | |
| Model 1 | 0.52 (0.44 - 0.62) | <0.001 |
| Model 2 | 0.62 (0.50 - 0.78) | <0.001 |
| Model 3 | 0.61 (0.48 - 0.78) | <0.001 |

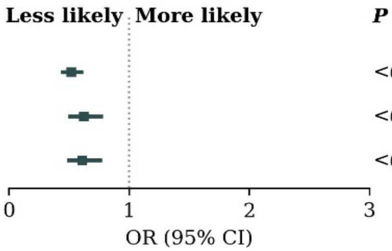


© 2025 Hulchafo II et al. *JAMA Network Open*.

## B. Length of Stay ≥ 3 days

### B-1. Asian/Pacific Islander (n=632)

| Language Category | Odds Ratio (95% Confidence Interval) | P value |
|---|---|---|
| **Marginalized language/identities** | | |
| Model 1 | 0.77 (0.50 - 1.20) | 0.25 |
| Model 2 | 0.76 (0.49 - 1.19) | 0.23 |
| Model 3 | 0.82 (0.52 - 1.30) | 0.40 |

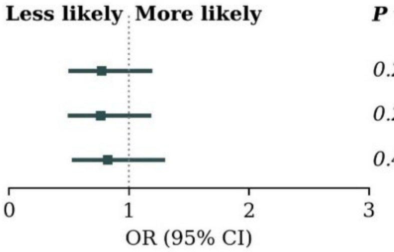


| Language Category | Odds Ratio (95% Confidence Interval) | P value |
|---|---|---|
| **Difficult patient** | | |
| Model 1 | 0.95 (0.79 - 1.16) | 0.64 |
| Model 2 | 0.96 (0.79 - 1.17) | 0.70 |
| Model 3 | 0.96 (0.78 - 1.19) | 0.73 |

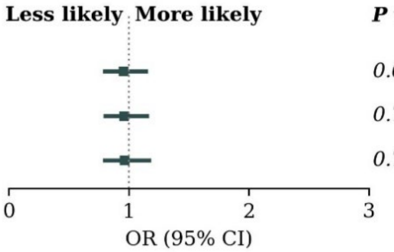


| Language Category | Odds Ratio (95% Confidence Interval) | P value |
|---|---|---|
| **Unilateral/authoritarian decisions** | | |
| Model 1 | 0.89 (0.72 - 1.09) | 0.24 |
| Model 2 | 0.88 (0.72 - 1.08) | 0.23 |
| Model 3 | 0.85 (0.69 - 1.06) | 0.14 |

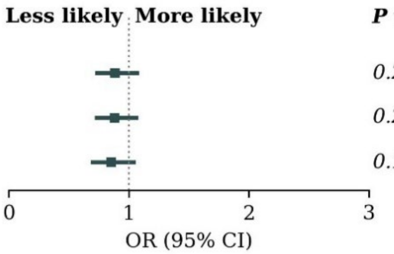


| Language Category | Odds Ratio (95% Confidence Interval) |
|---|---|
| **Questioning patient credibility** | -- |
| Model 1 | -- |
| Model 2 | -- |
| Model 3 | -- |

| Language Category | Odds Ratio (95% Confidence Interval) | P value |
|---|---|---|
| **Preferred language/ respecting patient autonomy** | | |
| Model 1 | 1.06 (0.89 - 1.26) | 0.52 |
| Model 2 | 1.07 (0.89 - 1.27) | 0.47 |
| Model 3 | 1.10 (0.91 - 1.32) | 0.32 |

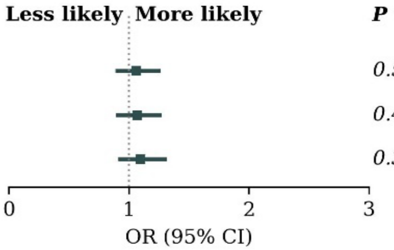


| Language Category | Odds Ratio (95% Confidence Interval) | P value |
|---|---|---|
| **Power/privilege** | | |
| Model 1 | 0.68 (0.50 - 0.91) | 0.009 |
| Model 2 | 0.69 (0.52 - 0.93) | 0.01 |
| Model 3 | 0.73 (0.54 - 0.99) | 0.04 |

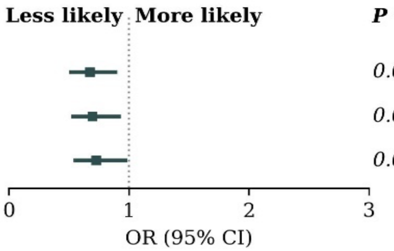


© 2025 Hulchafo II et al. *JAMA Network Open*.

## B-2. Black (n=1,236)

| Language Category | Odds Ratio (95% Confidence Interval) |
|---|---|
| **Marginalized language/identities** | |
| Model 1 | 2.37 (1.84 - 3.06) |
| Model 2 | 1.18 (0.88 - 1.57) |
| Model 3 | 1.17 (0.87 - 1.59) |

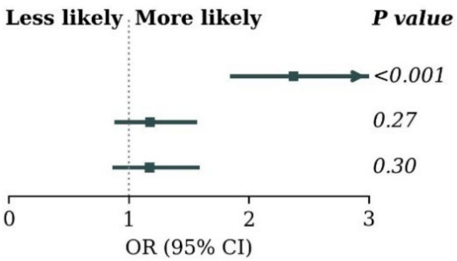


| Language Category | Odds Ratio (95% Confidence Interval) |
|---|---|
| **Questioning patient credibility** | |
| Model 1 | 0.49 (0.05 - 4.36) |
| Model 2 | 0.12 (0.01 - 1.27) |
| Model 3 | -- |

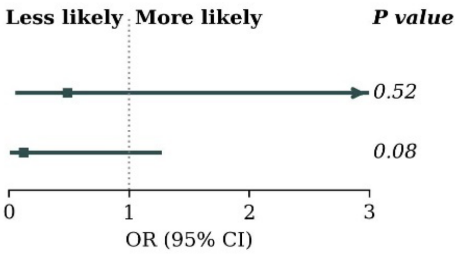


| Language Category | Odds Ratio (95% Confidence Interval) |
|---|---|
| **Difficult patient** | |
| Model 1 | 1.31 (1.13 - 1.52) |
| Model 2 | 1.25 (1.06 - 1.46) |
| Model 3 | 1.18 (0.99 - 1.39) |

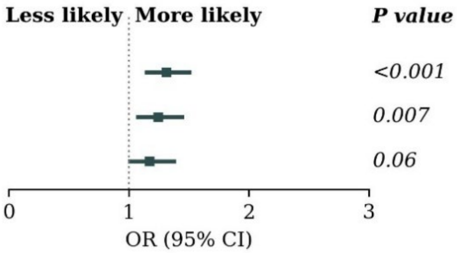


| Language Category | Odds Ratio (95% Confidence Interval) |
|---|---|
| **Preferred language/ respecting patient autonomy** | |
| Model 1 | 1.32 (1.14 - 1.51) |
| Model 2 | 1.19 (1.02 - 1.39) |
| Model 3 | 1.22 (1.04 - 1.42) |

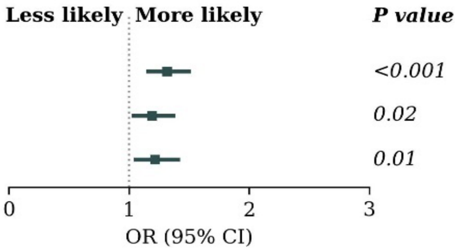


| Language Category | Odds Ratio (95% Confidence Interval) |
|---|---|
| **Unilateral/authoritarian decisions** | |
| Model 1 | 1.10 (0.94 - 1.28) |
| Model 2 | 1.10 (0.93 - 1.30) |
| Model 3 | 1.05 (0.89 - 1.26) |

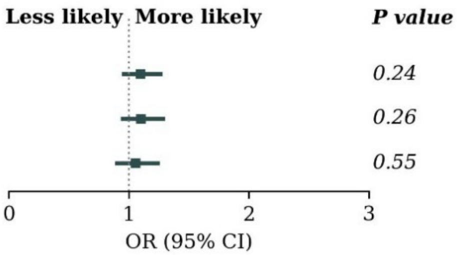


| Language Category | Odds Ratio (95% Confidence Interval) |
|---|---|
| **Power/privilege** | |
| Model 1 | 0.99 (0.81 - 1.21) |
| Model 2 | 1.00 (0.80 - 1.26) |
| Model 3 | 0.98 (0.77 - 1.24) |

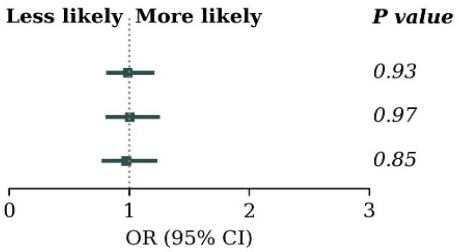


© 2025 Hulchafo II et al. *JAMA Network Open*.

**B-3. Hispanic (n=6,322)**

| Language Category | Odds Ratio (95% Confidence Interval) |
|---|---|
| **Marginalized language/identities** | |
| Model 1 | 2.17 (1.78 - 2.65) |
| Model 2 | 0.90 (0.69 - 1.16) |
| Model 3 | 0.96 (0.73 - 1.26) |

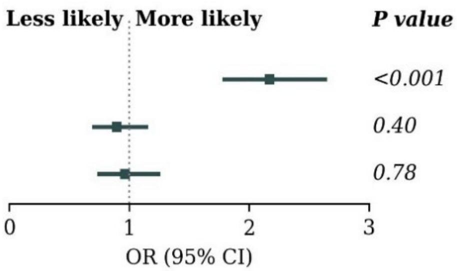


| Language Category | Odds Ratio (95% Confidence Interval) |
|---|---|
| **Questioning patient credibility** | |
| Model 1 | 0.67 (0.20 - 2.28) |
| Model 2 | 0.19 (0.04 - 0.93) |
| Model 3 | -- |

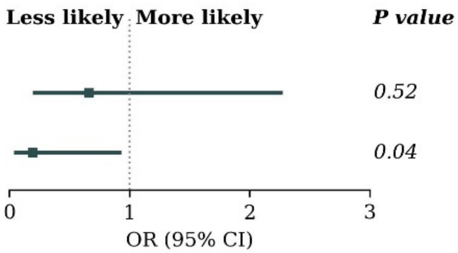


| Language Category | Odds Ratio (95% Confidence Interval) |
|---|---|
| **Difficult patient** | |
| Model 1 | 1.00 (0.90- 1.11) |
| Model 2 | 1.01 (0.88 - 1.16) |
| Model 3 | 1.00 (0.87 - 1.16) |

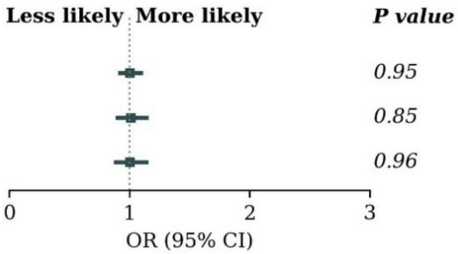


| Language Category | Odds Ratio (95% Confidence Interval) |
|---|---|
| **Preferred language/ respecting patient autonomy** | |
| Model 1 | 1.00 (0.91 - 1.10) |
| Model 2 | 0.93 (0.82 - 1.05) |
| Model 3 | 0.98 (0.86 - 1.11) |

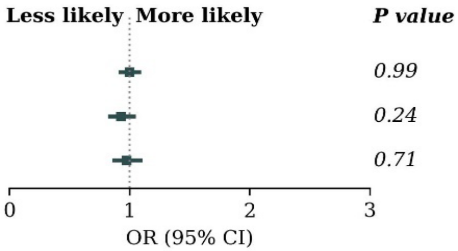


| Language Category | Odds Ratio (95% Confidence Interval) |
|---|---|
| **Unilateral/authoritarian decisions** | |
| Model 1 | 0.96 (0.86 - 1.07) |
| Model 2 | 0.97 (0.84 - 1.11) |
| Model 3 | 0.96 (0.83 - 1.11) |

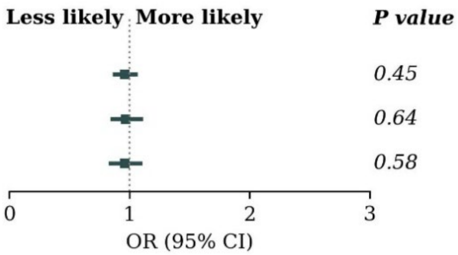


| Language Category | Odds Ratio (95% Confidence Interval) |
|---|---|
| **Power/privilege** | |
| Model 1 | 0.49 (0.42 - 0.57) |
| Model 2 | 0.62 (0.50 - 0.76) |
| Model 3 | 0.63 (0.51 - 0.78) |

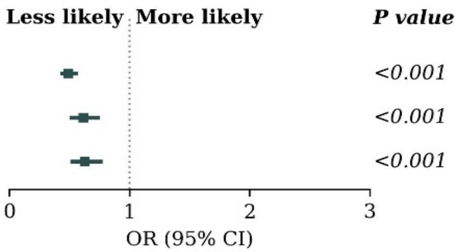


All models used White as a reference group as we assumed they would be least likely to experience stigmatizing language. Model 1: race and ethnicity (unadjusted). Model 2: Race and ethnicity + demographic characteristics: maternal age, marital status, insurance type, language. Model 3: Race and ethnicity, demographic and birth characteristics: parity, mode of birth, gestational age. Due to the low occurrence of questioning patient credibility category, we were not able to provide estimates.

Abbreviations: OR, Odds Ratio; CI, Confidence Intervals.

© 2025 Hulchafo II et al. *JAMA Network Open*.

**eFigure 2.** Unadjusted and Adjusted Logistic Regression Models Examining Any Stigmatizing or Positive Language by Patient Race and Ethnicity in the Full Study Sample (N=18,646)

| **Models** | **Any Stigmatizing or Positive Language Odds Ratio (95% Confidence Interval)** |
|---|---|
| **Asian/Pacific Islander** | |
| Model 1 | 1.01 (0.87 - 1.17) |
| Model 2 | 1.02 (0.89 - 1.19) |
| Model 3 | 0.99 (0.85 - 1.16) |
| **Black** | |
| Model 1 | 1.48 (1.3 - 1.68) |
| Model 2 | 1.31 (1.15 - 1.5) |
| Model 3 | 1.30 (1.13 - 1.5) |
| **Hispanic** | |
| Model 1 | 1.06 (0.98 - 1.15) |
| Model 2 | 0.97 (0.88 - 1.08) |
| Model 3 | 1.00 (0.89 - 1.11) |

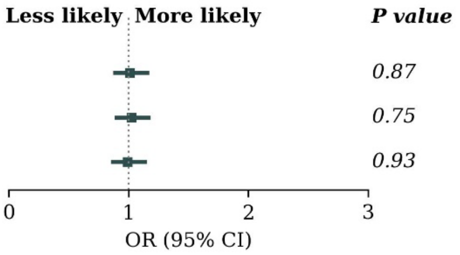


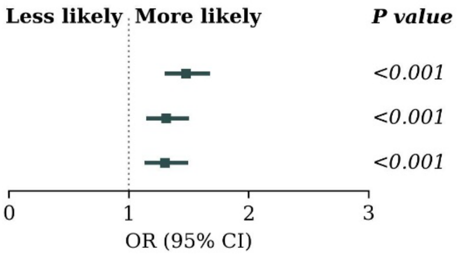


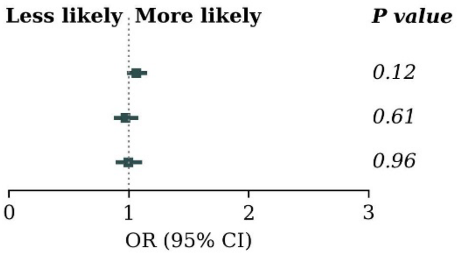


All models used White as a reference group as we assumed they would be least likely to experience stigmatizing language. Model 1: race and ethnicity (unadjusted). Model 2: Race and ethnicity + demographic characteristics: maternal age, marital status, insurance type, language. Model 3: Race and ethnicity, demographic and birth characteristics: parity, mode of birth, gestational age.

© 2025 Hulchafo II et al. *JAMA Network Open*.
